# The prevalence of dental caries among Egyptian children and adolescences and its association with age, socioeconomic status, dietary habits and other risk factors. A cross-sectional study

**DOI:** 10.12688/f1000research.17047.1

**Published:** 2019-01-03

**Authors:** Marwa M.S. Abbass, Sara Ahmed Mahmoud, Sara El Moshy, Dina Rady, Nermeen AbuBakr, Israa Ahmed Radwan, Attera Ahmed, Ahmed Abdou, Ayoub Al Jawaldeh

**Affiliations:** 1 Oral Biology Department, Faculty of Dentistry, Cairo University, Cairo, 11553, Egypt; 2 Pediatric Dentistry and Dental Public Health Department, Faculty of Dentistry, Cairo University, Cairo, 11553, Egypt; 3 Independent Researcher, Limerick, County Limerick, Ireland; 4 Biomaterials Department, Faculty of Dentistry,, Modern University for Technology and Information (MTI), Cairo, Egypt; 5 Cariology and Operative Department, Tokyo Medical and Dental University (TMDU), Tokyo, Japan; 6 Nutrition unit, World Health Organization Office for Eastern Mediterranean region , Cairo, Egypt

**Keywords:** Caries, Prevalence, Age, Socioeconomic, Dietary, Education, children, Adolescences

## Abstract

**Background**: Dental caries is a chronic, multifactorial disease, with limited data available for the Egyptian population. The aim of this study is to assess the prevalence of dental caries among Egyptian children and adolescents in correlation with age, gender, body mass index, socioeconomic status, parental education, biological risk factors and dietary habits.

**Methods**: A total number of 369 Egyptian children and adolescents (age ranges from 3-18 years) were examined over the period from 15 ^th^ November 2017 to 13 ^th^ January 2018. Socio-demographic data, oral hygiene measures and dietary habits for children were recorded. Dental status was analyzed using decayed, missing and filled tooth index (dmft) for deciduous dentition and (DMFT) index for permanent dentition. For mixed dentition (deft) index was used, d (decayed tooth indicated for filling), e (decayed tooth indicated for extraction) and f (filled tooth).

**Results**: 74% of the children had dental caries with mean dmft: 3.23±4.07; deft: 4.21±3.21; DMFT: 1.04±1.56. In primary dentition, dmft of the children was positively correlated with age, beans, candies, crackers, chocolates and inversely correlated with gender, socio-economic status (SES), parental education, brushing frequency of the parent, brushing frequency of the parent to the child teeth, brushing frequency of the child and consumption of eggs, fruits/vegetables, milk and milk products. In mixed dentition, deft was positively correlated with candies, crackers, citric juices, while negatively correlated with age, SES, parental education, brushing frequency of the parent to the child, brushing frequency of the child, fruits/vegetables. In permanent dentition, DMFT in children was positively correlated with age and chocolates while not correlated with any of the remaining risk factors.

**Conclusion**: The present study clarifies the significant risk factors associated with dental caries amongst Egyptian children. This will help in planning strategies to prevent and treat such disease.

## Introduction

Dental caries is a major public health issue and it is the most widespread chronic disease ^1^. Deciduous tooth decay was ranked as the 12 ^th^ most prevalent condition, affecting 560 million children in the 2015 Global Burden of Disease Study ^2^. Dental caries is a multifactorial disease, which can affect any age. It is highly related to and influenced by the patient’s dietary habits, sugar intake, salivary flow, salivary fluoride level and preventive behaviors. These factors, together with time, promote the microbial residence in the accumulated dental plaque to initiate dental caries ^1^.

In children, dental caries pattern depends on the timing of tooth eruption as well as harmful dietary habit. Therefore, age is considered as an important factor that affects dental caries prevalence in children ^3^. Although caries is common, parents are infrequently concerned about oral health measures and usually poor oral health is linked with low socio-economic status ^4^. Dental caries could only be prevented through addressing and changing the underlying etiological factors ^5, 6^.

It has been estimated that about 60% to 90% of children at school age suffer from this chronic ailment ^7, 8^. This percentage varies greatly in different population, with the incidence of dental caries in developing countries, including the Middle East, being much higher than its incidence in developed countries ^9, 10^.

Despite the high prevalence of dental caries in the Egyptian population, only a few epidemiological studies of dental caries among Egyptians have been published. Moreover, most of the available data, are grey literature which are not available on common search engines ^11– 15^. The most recently published epidemiological study on the oral health status in Egypt was held by WHO in collaboration with the Egyptian Ministry of Health in 2014 ^16^. Moreover, most of the epidemiological studies focused on children ^17, 18^ and only one focused on adolescents ^19^.

The prevalence of dental caries should be assessed continuously to plan and implement an efficient children oral health agenda and awareness programs for parents and school teachers in order to improve oral health. Therefore, this study was conducted to elucidate the prevalence of dental caries among Egyptian children and adolescence in correlation with age, socioeconomic level, dietary habits, oral hygiene measures and body mass index.

## Subjects and methods

This study was carried out according to the regulations of the Research Ethics Committee of the Faculty of Dentistry, Cairo University, Egypt [Approval: 171217]. Written informed consent was obtained from children’s parents or guardians to participate in the study. Verbal consent was taken from adolescents in addition to written consent from their parent/guardian. The subjects in this study were recruited from the outpatients' clinics of Faculty of Dentistry, Cairo University and from two private nurseries in Nasr City and Maadi from 15th November 2017 to 13th January 2018. The inclusion criteria were; Age: starting from 3 years to 12 years for the children group, from 13 years to 18 years for adolescence; Gender: Males & Females; Ethnicity: Egyptians. The exclusion criteria were; Previous history/current radiotherapy and/or chemotherapy; Subjects undergoing orthodontic therapy, which might preclude normal tooth brushing; Subjects who, in the opinion of the Investigator, may be non-compliant with study procedures.

### Sample size calculation

$$\mathrm{According}\mathrm{to}\mathrm{the}\mathrm{following}\mathrm{simple}\mathrm{formula}\;\;\;\;\;\;\;n^{\prime }=\frac{NZ^{2}P(1-P)}{d^{2}(N-1)+Z^{2}P(1-P)}$$

^20^

n' = sample size with finite population correction, N = children and adolescents population size (50,000,000), Z = Z statistic for a level of confidence which is conventional, Z value is (1.96). P = Expected prevalence (51.4%–70%) and d = Precision (5%, d = 0.05), the sample size for caries in children and adolescents was estimated 369. The prevalence of dental caries in Egypt was estimated to be 60% according to WHO, 2014 ^16^, Hamila, 2014 ^18^ reported ~70%, Mubarak *et al*. 2011 ^19^ reported 51.4% and Abou El yazeed *et al*., 2011 ^17^ reported 60.4%. The prevalence of dental caries in India was 61.4% in adolescent ^21^ and in Australia was 51.6% among different refugee ^22^.

### Data collection and grouping

The socio-demographic data collected by the authors included; name, age, gender, address, education (governmental, experimental or private), number of family members and their guardians' occupation and level of education in addition to oral hygiene measures for the children and their guardians as well as the dietary habits through a questionnaire. (Extended data ^90^)

Children were classified according to their age into 5 groups; group I (3–5 years old); group II (5–7 years old); group III (7– 9); group IV (9– 12); group V (12– 18). Moreover, they were categorized based on their socioeconomic status, into low, moderate and high groups based on (level of education and its type, guardians' occupation and address) ^25^.

A Beurer scale (Ulm, Germany) was used to measure weights with individuals wearing clothing but no shoes. Standing heights were measured to the nearest 0.1cm using a stadiometer according to WHO, 1995 ^23^. Body mass index (BMI) thresholds were calculated from the measured height and weight ^24, 25^. The obtained BMI values were plotted on age and gender–specific percentiles given by the Centers for Disease Control and Prevention ^26^. Children were categorized into four groups based on their BMI percentiles; underweight (<5th percentile), normal group (≥5th - <85th percentile), overweight (≥85th - <95th percentile) and obese (≥95th percentile).

### Oral examination

Prior to beginning of examination, authors (M.M.S., S.A.M, S.E., D.R., N.A., I.A.R.) were trained and caliberated over 3 sessions over 3 days. Differences in observations were discussed among the examiners for reassessment and to reach a consensus ^19, 27^. Oral examination was carried out according to WHO criteria ^28^ on a dental chair in artificial light by using a plain mouth mirror and a dental probe. All present teeth were taken into consideration during the clinical examination ^27^.

A tooth was considered carious when, any lesion in a pit or fissure or on a smooth tooth surface had a detectably softened floor, undermined enamel or softened wall, tooth surface containing temporary filling requiring further treatment and when a tooth surface containing a permanent restoration with an area of decay (either primary or secondary caries). Caries severity was measured for permanent teeth by DMFT index, which records the number of D (decayed tooth), M (missing tooth) and F (filled tooth). For primary teeth the dmft index was used; d (decayed teeth, m (missed teeth) and f (filled tooth). For mixed dentition deft index was used; d (decayed tooth indicated for filling), e (decayed tooth indicated for extraction) and f (filled tooth) ^29^.

### Statistical analysis

The statistical analysis was performed using R statistical package, version 3.3.1 (2016-06-21). For descriptive analyses, variables were described in terms of means, standard deviations (SD), medians and ranges. Shapiro-Wilk test for normality showed that all the studied parameters were not normally distributed. For comparative analysis, the non-parametric Kruskal-Wallis test was performed. Spearman’s Correlation Coefficient was calculated for correlation analysis. Results were considered significant at a P value of ≤ 0.05.

## Results

### I- Population profile

The mean dmft, deft, DMFT, age (years) and BMI (kg/m ^2^) for the whole sample were (3.23±4.07, 4.21±3.21, 1.04 ±1.56, 7.2±3.53, 18.19±3.9) respectively. The number and percentage of children in different categories in each studied parameter as well as comparisons between them are presented in Table 1. Different categories within each parameter were statistically significant in correlation to each other (p-value <0.05) except for the variable “Reason for the children not brushing their teeth”.

**Table 1.** Descriptive analysis of categorical variables and comparisons between proportions- N=369.

Parameter	Categories number (%)						Pearson’s Chi-squa. test	
							*Χ* ^2^	p-value
**1- Age**	**AI (3–4 years)**	**AII (5–6 years)**	**AIII (7–8 years)**	**AIV (9–11 years)**	**AV (12–17 years)**		33.32	<0.0001 *
	113 (30.62)	81 (21.95)	56 (15.18)	51 (13.82)	68 (18.43)			
**2- Gender**	**Males**			**Females**			8.2	0.0042 *
	157 (42.55)			212 (57.45)				
**3- Body Mass Index**	**Underweight (<5)**	**Normal (≥5–<85)**	**Overweight (≥85–<95)**		**Obese (≥ 95)**		168.85	<0.0001 *
	26 (7.16)	190 (52.34)	55 (15.15)		92 (25.34)			
**4- SES**	**Low**		**Moderate**		**High**		29.94	<0.0001 *
	162 (44.02)		129 (35.05)		77 (20.92)			
**5- Parental education**	**Low**		**Moderate**		**High**		38.14	<0.0001 *
	76 (20.82		117 (32.05)		172 (47.12)			
**6- Biological Risk Factors**	**No brushing**	**Infrequent**	**once daily**	**Twice daily**	**Three times**	**Other**		
** Mother/Father own brushing** **frequency**	63 (17.07)	102 (27.64)	100 (27.1)	72 (19.51)	30 (8.13)	1 (0.27)	128.6	<0.0001 *
** Mother/Father brushing for the** **child**	99 (33.11)	76 (25.42)	61 (20.4)	37 (12.37)	21 (7.02)	5 (1.67)	125.07	<0.0001 *
** Child’s own brushing frequency**	69 (20.23)	102 (29.91)	78 (22.87)	64 (18.77)	22 (6.45)	6 (1.76)	114.1	<0.0001 *
** Reason for no brushing for the** **child**	**I don't know how to brush**	**I don't have time**	**I don't have dental cleaning devices** **(ie. toothbrush etc.)**		**Other**			
	35 (22.44)	71 (45.51)	42 (26.92)		8 (5.12)		51.538	<0.0001 *
** Reason if child does not brush**	**I don't know how to brush**		**I forget**		**Other**			
	43 (31.85)		55 (40.74)		37 (27.4)		3.7333	0.1546
**7-Dietary Habits**	**≤ 2 times/week**		**3–6 times/week**		**1–6 times/day**			
** Bread**	36 (9.76)		3 (0.81)		330 (89.43)		526.98	<0.0001 *
** Other carbohydrates**	68 (18.58)		83 (22.68)		215 (58.74)		107.26	<0.0001 *
** Eggs**	186 (50.41)		80 (21.68)		103 (27.91)		50.55	<0.0001 *
** Fruits/Vegetables**	89 (24.18)		106 (28.8)		173 (47.01)		32.16	<0.0001 *
** Milk**	128 (34.69)		44 (11.92)		197 (53.39)		95.46	<0.0001 *
** Milk products**	98 (26.56)		54 (14.63)		217 (58.81)		115.63	<0.0001 *
** Beans**	168 (45.65)		28 (7.61)		172 (46.74)		109.65	<0.0001 *
** Jam, Molasses and Honey**	233 (63.14)		49 (13.28)		87 (23.58)		153.43	<0.0001 *
** Candies**	114 (30.89)		32 (8.67)		223 (60.43)		149.28	<0.0001 *
** Crackers**	120 (32.6)		30 (8.15)		218 (59.24)		144.15	<0.0001 *
** Junk food**	304 (82.6)		16 (4.35)		48 (13.04)		406.26	<0.0001 *
** Chocolate**	195 (53)		52 (14.13)		121 (32.88)		83.39	<0.0001 *
** Soda**	242 (65.94)		40 (10.9)		85 (23.16)		183.86	<0.0001 *
** Juices**	167 (45.26)		45 (12.2)		157 (42.55)		74.6	<0.0001 *
** Citric juices**	280 (75.88)		44 (11.92)		45 (12.2)		300.6	<0.0001 *
** Caffeinated drinks**	200 (54.01)		9 (2.44)		160 (43.36)		164.99	<0.0001 *

*Statistical significance at p-value ≤ 0.05.

### II- dmft and different caries risk factors ( Table 2, Figure 1)

Age was positively correlated with dmft (Spearman’s rho=0.32, p-value<0.0001). The highest mean dmft was for children aged between 5 to 6 years old (5.62 ±4.27) with a statistically significant difference between medians (p-value=0.0006).

**Table 2.** dmft and different risk factors - N= 369.

Variables	Categories - Mean (SD) / Median										Correlation		K-W test
**1- Age**	**AI (3–4 years)**		**AII (5–6 years)**		**AIII (7–8 years)**		**AIV (9–11 years)**		**AV (12–17 years)**		**rho**	**p-value**	**p-value**
	2.42 (3.71)	0 (0-16)	5.62(4.27)	4.5(0-18)	2.25(2.63)	2(0-5)	NA	NA	NA	NA	0.32	<0.0001 *	0.0006 *
**2- Gender**	**Males**					**Females**							
	3.88(4.4)			3 (0-18)			2.68 (3.72)		1 (0-16)		-0.16	0.0483 *	0.0486 *
**3- Body Mass Index**	**Underweight (<5)**		**Normal (≥5–<85)**		**Overweight (≥85–<95)**			**Obese (≥ 95)**					
	4.18(5.84)	0 (0-16)	3 (4.24)	1 (0-18)	2.87 (3.66)	1 (0-13)	3.48 (3.67)		3(0-12)		0.09	0.2742	0.6462
**4- SES**	**Low**			**Moderate**			**High**						
	5.32 (3.63)	5 (0-16)	4.5 (4.99)		3 (0-18)		0.52 (1.4)		0 (0-8)		-0.64	<0.0001 *	<0.0001 *
**5- Parental Education**	**Low**			**Moderate**			**High**						
	6.47(4.44)	6 (0-16)	4.47 (3.94)		4 (0-18)		1.41 (2.89)		0 (0-13)		-0.57	<0.0001 *	<0.0001 *
**6- Biological Risks**	**No brushing**		**Infrequent**		**Once daily**		**Twice daily**		**Three times**				
** -Parent own brushing**	6 (5.4)	5 (0-16)	6.44(4.33)	6.5 (0-18)	2.06 (2.3)	1 (0-6)	1.64(2.39)	0 (0-10)	0.6(1.89)	0 (0-9)	-0.5	<0.0001 *	<0.0001 *
** -Parent brushing for** **child**	5.18(4.72)	4.5(0-16)	5.97(4.19)	5 (0-18)	1.51 (2.3)	0 (0-8)	2.04(3.22)	0 (0-10)	0.06(0.24)	0 (0-1)	-0.5	<0.0001 *	<0.0001 *
** -Child own brushing**	4.58(4.57)	4 (0-16)	4.63(4.39)	4 (0-18)	2.65(3.15)	2 (0-10)	0.94(2.04)	2 (0-8)	0.06(0.24)	0 (0-1)	-0.47	<0.0001 *	<0.0001 *
**7- Dietary Habits**	**≤ 2 times/week**			**3–6 times/week**			**1–6 times/day**						
** -Bread**	2.56 (4.5)	0 (0-16)	4.5 (6.36)		4.5 (0-9)		3.28 (4.02)		2 (0-18)		0.08	0.317	0.5142
** -Other carbohydrates**	4.62 (5.62)	2.5 (0-18)	1.96 (2.71)		0 (0-9)		3.4 (3.92)		2 (0-16)		0.04	0.576	0.0548
** -Eggs**	4.55 (4.44)	4 (0-18)	2.3 (3.45)		1 (0-14)		2.75 (3.92)		0 (0-16)		-0.21	0.0089 *	0.0077 *
** -Fruits/Vegetables**	5.71 (4.55)	5 (0-16)	2.61 (3.88)		1 (0-18)		2.61 (3.64)		1 (0-14)		-0.22	0.0046 *	0.0006 *
** -Milk**	4.87 (4.35)	4 (0-16)	4.33 (4.44)		4 (0-18)		2.38 (3.66)		0 (0-16)		-0.31	<0.0001 *	0.0005 *
** -Milk products**	5.11 (4.74)	4 (0-16)	2.35 (2.99)		1 (0-9)		2.71 (3.82)		1 (0-18)		-0.2	0.0093 *	0.008 *
** -Grains**	2.7 (4)	0 (0-18)	4.6 (4.5)		4 (0-16)		3.64 (4.01)		3 (0-16)		0.18	0.0206 *	0.0366 *
** -Jam, Molasses,** ** Honey**	3.46 (4.32)	2 (0-18)	1.48 (2.69)		0 (0-8)		3.97 (4.04)		4 (0-14)		0.04	0.9561	0.0073 *
** -Candies**	1.86 (3.05)	0 (0-12)	3.72 (4.38)		2 (0-13)		4.21 (4.45)		3.5 (0-18)		0.29	0.0002 *	0.0012 *
** -Crackers**	1.8 (3.08)	0 (0-13)	2 (2.45)		1.5 (0-7)		4.3 (4.49)		4 (0-18)		0.3	0.0001 *	0.0008 *
** -Junk food**	2.99 (3.76)	1 (0-16)	3 (NA)		3 (3-3)		5.11 (5.85)		3.5 (0-18)		0.1	0.2092	0.45
** -Chocolate**	2.03 (3.18)	0 (0-13)	3.5 (2.78)		4 (0-8)		5.02 (5.02)		4 (0-18)		0.34	<0.0001 *	<0.0001 *
** -Soda drinks**	2.97 (4)	1 (0-18)	4.25 (3.28)		4.5 (0-12)		3.54 (4.54)		1 (0-16)		0.07	0.413	0.1315
** -Juices**	3.35 (4.03)	2 (0-18)	3.52 (4.63)		1 (0-14)		3.01 (3.97)		1 (0-16)		-0.04	0.5759	0.8527
** -Citric juices**	3.19 (3.92)	2 (0-18)	2.75 (3.87)		0 (0-12)		3.81 (4.93)		1 (0-16)		-0.01	0.9162	0.6387
** -Caffeinated drinks**	3.13 (3.61)	2 (0-16)	5 (NA)		5 (NA)		3.32 (4.56)		1 (0-18)		-0.04	0.6431	0.6342

The correlation coefficient, rho, ranges from -1 to +1. Where 1= perfect positive correlation, 0=no correlation, -1 = perfect negative (inverse) correlation. *Statistical significance at p-value ≤ 0.05

**Figure 1.**
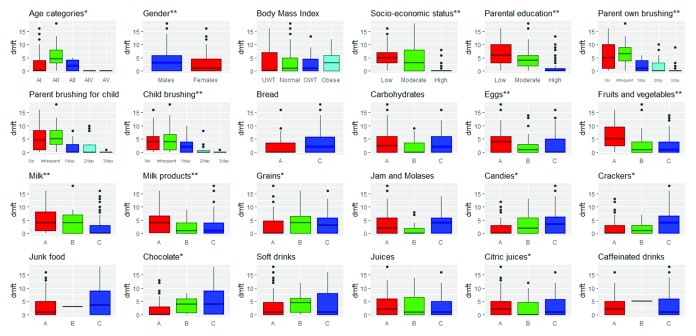
dmft and different risk factors - N= 369: *+ve correlations; ** inverse correlations; A *: ≤ 2 times/week* B *: 3–6 times/week* C *: 1–6 times/day*.

Gender, SES and parental educational were inversely correlated with dmft (Spearman’s rho=-0.16, -0.64, -0.57, p-value=0.0483, <0.0001, <0.0001) respectively. Male children, children with low SES and children with low level of parental education had the highest mean dmft (3.88 ±4.4, 5.32 ±3.63, 6.47±4.44) with statistically significant difference (p-value= 0.0486, <0.0001, <0.0001) respectively.

BMI was not correlated with dmft (Spearman’s rho=0.09, p-value=0.2742) and the highest mean dmft was for underweight children (4.18 ±4.27). The difference in medians was statistically insignificant (p-value >0.05).

dmft of children increased with decreased brushing frequency of parents, brushing frequency of parents to their children and children’s own brushing frequency (Spearman’s rho= - 0.5, p-value<0.0001) and the highest mean dmft were with infrequent brushing (6.44 ±4.33, 5.97 ±4.19, 4.63 ±4.39) respectively. The difference in medians was statistically significant (p-value<0.0001).

Positive correlations exist between dmft and the consumption of candies, crackers, chocolate and beans (rho=0.29, 0.3, 0.34, 0.18, p-value=0.0002, 0.0001, <0.0001, 0.0206) with highest mean dmft with frequency 1–6 times per day for candies, crackers, chocolate (4.21±4.45, 4.3±4.49, 5.02 ±5.02) respectively and 3–6 times per week for beans (4.6±4.5). Negative correlations occur between dmft and the consumption of eggs, fruits/vegetables, milk and milk products (rho= -0.21, -0.22, -0.31, -0.2, p-value=0.0089, 0.0046, <0.0001, 0.0093) with highest mean dmft with frequency ≤ 2 times/week (4.55±4.44, 5.71±4.55, 4.87 ±4.35, 5.11 ±4.74) respectively. No correlation was found between dmft and the consumption of bread, other carbohydrates, jams, junk food, soda, juices, citric juices and caffeinated drinks (p-value>0.05).

### III- deft and different caries risk factors ( Table 3, Figure 2)

The highest mean deft was for children aged between 5 to 6 years old, children with low SES and children of patients with low level of parental education (5.51 ±3.28, 4.68 ±3.24, 4.62 ±3.25). The differences in medians were statistically significant for age and SES (p-value= 0.0013, 0.0277), while borderline insignificant for parental education (p-value=0.0542). Age, SES and parental education were inversely correlated with deft in children (Spearman’s rho= -0.42, -0.19, -0.16, p-value<0.0001, 0.0123, 0.0354).

**Table 3.** deft and different risk factors - N= 369.

**Variables**	**Categories - Mean (SD) / Median**										**Correlation**		**K-W test**
**1- Age**	**AI (3–4 years)**		**AII (5–6 years)**		**AIII (7–8 years)**		**AIV (9–11 years)**		**AV (12–17 years)**		**rho**	**p-value**	**p-value**
	4 (NA)	4 (NA)	5.51(3.28)	5 (0-14)	5.22 (3.24)	4.5(0-13)	3.06 (2.5)	3 (0-12)	1.95 (2.5)	1 (0-9)	-0.42	<0.0001 *	0.0013 *
**2- Gender**	**Males**					**Females**							
	4.04(2.93)		4 (0-14)		4.34 (3.41)			4 (0-13)			0.01	0.8758	0.8752
**3- Body Mass Index**	**Underweight (<5)**		**Normal (≥5–<85)**		**Overweight (≥85–<95)**			**Obese (≥ 95)**					
	3.67(2.27)	4 (0-7)	4.19 (3.13)	4 (0-13)	4.73 (3.65)	4.5 (0-14)	4.19 (3.33)		4 (0-12)		0.03	0.7132	0.9166
**4- SES**	**Low**			**Moderate**			**High**						
	4.68 (3.24)	4 (0-13)	3.78 (2.9)		4 (0-13)		2.81 (4.14)		1 (0-14)		-0.19	0.0123 *	0.0277 *
**5- Parental Education**	**Low**			**Moderate**			**High**						
	4.62 (3.25)	4 (0-12)	4.49 (2.95)		4 (0-13)		3.55 (3.47)		3 (0-14)		-0.16	0.0354 *	0.0542
**6- Biological Risks**	**No brushing**		**Infrequent**		**Once daily**		**Twice daily**		**Three times**				
** -Parent own** ** brushing**	4.65(2.75)	4(0-13)	4.5(3.12)	4(0-13)	3.38(3.31)	3(0-13)	4.29(3.95)	3(0-14)	4.5 (2.89)	4.5(1-8)	-0.14	0.0815	0.2237
** -Parent brushing** ** for child**	5.3 (3.34)	5 (0-13)	4.1(2.75)	4 (0-12)	4.88(3.84)	4.5(0-14)	3.3 (2.75)	3 (0-8)	3 (2.65)	4 (0-5)	-0.17	0.0456 *	0.3299
** -Child own** ** brushing**	4.91(2.62)	5 (0-10)	4.25(3.01)	4 (0-14)	4.1 (3.45)	4 (0-13)	1.29(1.33)	1 (0-4)	1.67(2.89)	0 (0-5)	-0.31	0.0001 *	0.0003 *
**7- Dietary Habits**	**≤ 2 times/week**			**3–6 times/week**			**1–6 times/day**						
** -Bread**	3.3 (2.98)	2.5 (0-13)		10 (2.83)		10 (8-12)		4.26 (3.17)		4 (0-14)	0.06	0.4074	0.0484 *
** -Other** ** carbohydrates**	4.44 (2.8)	4 (0-11)		4.12 (2.65)		4 (0-12)		4.15 (3.55)		3 (0-14)	-0.08	0.285	0.5628
** -Eggs**	4.18 (2.99)	4 (0-13)		3.77 (3.61)		3 (0-13)		4.71 (3.49)		4 (0-14)	0.01	0.8893	0.3922
** -Fruits/Vegetables**	5.6 (3.2)	5 (0-13)		4.05 (2.69)		4 (0-10)		3.09 (3.12)		2 (0-14)	-0.36	<0.0001 *	<0.0001 *
** -Milk**	4.43 (3.19)	4 (0-13)		5.3 (2.6)		5 (0-9)		3.49 (3.33)		3 (0-14)	-0.12	0.119	0.0171 *
** -Milk products**	4.59 (2.8)	4 (0-12)		4.39 (3.19)		4.5 (0-12)		3.89 (3.48)		3 (0-14)	-0.15	0.0527	0.1501
** -Grains**	4.01 (3.11)	4 (0-14)		4.5 (3.06)		4 (0-10)		4.35 (3.37)		4 (0-13)	0.04	0.6093	0.7777
** -Jam, Molasses,** ** Honey**	4.38 (3.37)	4 (0-14)		3.88 (2.78)		3 (0-10)		3.96 (3.02)		4 (0-13)	-0.05	0.5366	0.8115
** -Candies**	2.96 (2.93)	2 (0-12)		3.47 (2.38)		3 (0-9)		4.86 (3.27)		4 (0-14)	0.29	0.0001 *	0.0009 *
** -Crackers**	2.22 (2.77)	2 (0-14)		3.73 (1.56)		4 (1-6)		4.77 (3.23)		4 (0-13)	0.34	<0.0001 *	<0.0001 *
** -Junk food**	4.06 (3.27)	4 (0-14)		4.77 (3.22)		4 (1-12)		4.78 (2.89)		5 (0-10)	0.13	0.1046	0.2675
** -Chocolate**	3.89 (3.19)	3 (0-13)		4.58 (3.53)		4 (0-14)		4.42 (3.15)		4 (0-13)	0.09	0.2277	0.4079
** -Soda drinks**	4.21 (3.18)	4 (0-13)		4.9 (3.97)		5 (0-14)		3.92 (2.86)		4 (0-10)	-0.01	0.9181	0.6463
** -Juices**	3.73 (2.68)	3 (0-12)		4.28 (2.39)		4 (0-8)		5.02 (4.06)		4.5 (0-14)	0.14	0.0823	0.2094
** -Citric juices**	3.9 (3.03)	4 (0-14)		4.27 (2.91)		4 (0-10)		6.32 (3.96)		7 (0-13)	0.18	0.019 *	0.0339 *
** -Caffeinated drinks**	4.17 (3.13)	4 (0-13)		2.6 (3.58)		0(0-7)		4.43 (3.34)		4 (0-14)	0.04	0.6383	0.4162

*The correlation coefficient, rho, ranges from -1 to +1. Where 1= perfect positive correlation, 0=no correlation, -1 = perfect negative (inverse) correlation.* *Statistical significance at p-value ≤ 0.05.

**Figure 2.**
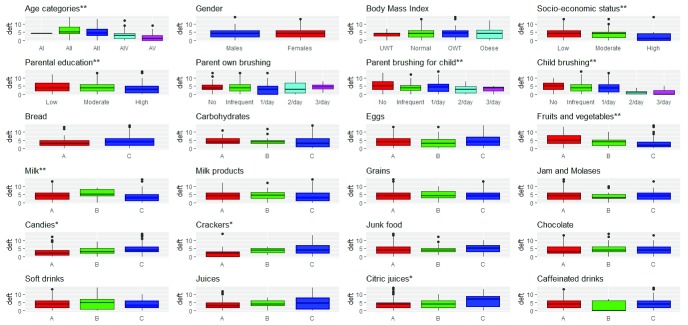
deft and different risk factors - N= 369: *+ve correlations; ** inverse correlations; A *: ≤ 2 times/week* B *: 3–6 times/week* C *: 1–6 times/day*.

The highest mean deft was for females (4.34 ±3.41) and overweight children (4.73 ±3.65). The difference in medians was statistically insignificant (p-value>0.05) and correlation coefficients revealed no correlation between deft and either gender or BMI (Spearman’s rho=0.01, 0.03, p-value=0.8758, 0.7132).

The highest mean deft was for children whose parents don’t brush their own teeth (4.65 ±2.75), or their children teeth (5.3 ±3.34), or children who don’t brush their own teeth (4.91 ±2.62). The difference in medians were statistically insignificant for parents own brushing frequency and brushing frequency to their children’s teeth (p-value>0.05), while it was statistically significant for children’s own brushing frequency (p-value= 0.0003). The correlation coefficient revealed no correlation between deft and parents own brushing frequency (rho= -0.14, p-value=0.0815), while inverse correlation with parents brushing frequency to their children’s teeth and children own brushing frequency (rho= -0.17, -0.31, p-value=0.0456, 0.0001).

The highest mean deft was for children who consume candies, crackers, junk food, juices, citric juices and caffeinated drinks 1–6 times per day (4.86±3.27, 4.77±3.23, 4.78± 2.89, 5.02±4.06, 6.32±3.96, 4.43±3.34) and children who consume bread, beans, chocolates and soda 3–6 times per week (10±2.83, 4.5±3.06, 4.58±3.53, 4.9±3.97), as well as children who consume other carbohydrates and jams less than or equal to two times a week (4.44 ±2.8, 4.38±3.37). The lowest mean deft was for children who consume eggs 3-6 times per week (3.77 ±3.61) and children who consume fruits/vegetables, milk and milk products 1–6 times per day (3.09 ±3.12, 3.49 ±3.33, 3.89±3.48) respectively.

The differences in medians for deft with fruits/vegetables, candies, crackers and citric juices consumption were statistically significant. There were positive correlations between candies, crackers, citric juices consumption and deft of children (rho=0.29, 0.34, 0.18, p-value=0.0001, <0.0001, 0.019), while there was inverse correlation between fruits/vegetables and deft (rho= -0.36, p-value <0.0001). No correlation was detected between deft of children and the consumption of bread, other carbohydrates, eggs, milk, milk products, beans, jams, junk food, chocolates, soda, juices and caffeinated drinks (p-value>0.05).

### III- DMFT and different caries risk factors ( Table 4, Figure 3)

The highest mean DMFT was for adolescents aged between 12 to 17 years (1.68 ±1.92). The difference in medians was statistically significant (p-value= <0.0001). Age was positively correlated with DMFT (Spearman’s rho=0.36, p-value<0.0001).

**Table 4.** DMFT and different risk factors - N= 369.

Variables	Categories - Mean (SD) / Median										Correlation		K-W test
**1- Age**	**AI (3–4 years)**		**AII (5–6 years)**		**AIII (7–8 years)**		**AIV (9–11 years)**		**AV (12–17 years)**		**rho**	**p-value**	**p-value**
	NA	NA	0.32 (0.9)	0 (0-4)	0.59 (1)	0(0-4)	1.2 (1.55)	0(0-5)	1.68 (1.92)	1(0-8)	0.36	<0.0001 *	<0.0001 *
**2- Gender**	**Males**					**Females**							
	1.28 (1.9)		0(0-8)		0.88 (1.28)			0 (0-5)			-0.05	0.4363	0.435
**3- Body Mass Index**	**Underweight (<5)**		**Normal (≥5–<85)**		**Overweight (≥85–<95)**			**Obese (≥ 95)**					
	1 (2.33)	0(0-8)	1.15 (1.52)	0 (0-6)	0.53 (1.34)	0(0-6)	1.02 (1.4)		0(0-5)		-0.04	0.5794	0.0353 *
**4- SES**	**Low**			**Moderate**			**High**						
	1.13 (1.67)	0(0-8)	0.92 (1.42)		0 (0-6)		1.29 (1.73)		0 (0-5)		-0.02	0.7829	0.7764
**5- Parental Education**	**Low**			**Moderate**			**High**						
	1.45 (1.93)	1(0-8)	0.89 (1.43)		0 (0-6)		0.93 (1.42)		0(0-6)		-0.07	0.2968	0.2141
**6- Biological Risks**	**No brushing**		**Infrequent**		**Once daily**		**Twice daily**		**Three times**				
** -Parent own brushing**	1.28(1.78)	0 (0-5)	1.1(1.59)	0 (0-6)	0.75(1.14)	0(0-6)	1.19(1.57)	0 (0-5)	1.6 (3.58)	1(0-8)	-0.03	0.648	0.8016
** -Parent brushing for** **child**	1.11(1.58)	0 (0-5)	0.88(1.53)	0 (0-6)	1.19(1.86)	0 (0-8)	1.18(1.78)	1 (0-6)	0 (0)	0 (0-0)	-0.02	0.8413	0.6872
** -Child own brushing**	1.19(1.47)	1 (0-4)	1.31(1.85)	0 (0-8)	1 (1.67)	0 (0-6)	0.85(1.25)	0 (0-5)	1.25 (1.5)	1 (0-3)	-0.08	0.2909	0.7799
**7- Dietary Habits**	**≤ 2 times/week**			**3–6 times/week**			**1–6 times/day**						
**-Bread**	1.25 (1.83)		0 (0-5)		1 (1.41)		1 (0-2)		1.02 (1.53)	0 (0-8)	-0.02	0.7909	0.957
**-Other** **carbohydrates**	1.12 (1.55)		0 (0-5)		1.32 (1.95)		0 (0-8)		0.93 (1.43)	0 (0-6)	-0.05	0.4446	0.677
**-Eggs**	1.07 (1.52)		0 (0-8)		1.18 (1.78)		0 (0-6)		0.77 (1.44)	0 (0-5)	-0.09	0.1799	0.307
**-Fruits/Vegetables**	1.23 (1.68)		0 (0-6)		1.08 (1.9)		0 (0-8)		0.9 (1.27)	0 (0-5)	-0.03	0.6737	0.6704
**-Milk**	1.15 (1.55)		0 (0-6)		0.96 (2.06)		0 (0-8)		0.95 (1.43)	0 (0-6)	-0.05	0.4692	0.3403
**-Milk products**	1.19 (1.79)		0 (0-8)		1.13 (1.61)		0 (0-5)		0.93 (1.41)	0 (0-6)	-0.04	0.592	0.8569
** -Grains**	0.93 (1.53)		0 (0-6)		1.57 (2.47)		0 (0-8)		1.06 (1.44)	0 (0-5)	0.08	0.2462	0.4485
** -Jam, Molasses,** **Honey**	1.06 (1.6)		0 (0-8)		1.3 (1.72)		0.5 (0-6)		0.86 (1.37)	0 (0-5)	-0.02	0.7349	0.5833
** -Candies**	0.86 (1.54)		0 (0-5)		1.38 (2.16)		0 (0-8)		1.05 (1.46)	0 (0-6)	0.08	0.2202	0.2972
** -Crackers**	0.73 (1.08)		0 (0-4)		1.59 (2.43)		0 (0-8)		1.22 (1.6)	0 (0-6)	0.06	0.4088	0.5112
** -Junk food**	1.05 (1.61)		0 (0-8)		0.87 (1.3)		0 (0-4)		0.93 (1.31)	0 (0-5)	-0.01	0.9314	0.9739
** -Chocolate**	0.83 (1.48)		0 (0-8)		1.18 (1.78)		0 (0-6)		1.3 (1.56)	1 (0-6)	0.16	0.0178 *	0.0584
** -Soda drinks**	0.98 (1.54)		0 (0-8)		1.25 (1.75)		0 (0-5)		1.09 (1.55)	0 (0-6)	0.05	0.5166	0.7974
** -Juices**	0.98 (1.52)		0 (0-6)		1.86 (2.23)		1.5 (0-8)		0.9 (1.34)	0 (0-6)	0.02	0.7458	0.1589
** -Citric juices**	1.03 (1.51)		0 (0-6)		1.4 (2.23)		0 (0-8)		0.68 (1.11)	0 (0-4)	-0.03	0.6481	0.7295
** -Caffeinated drinks**	1 (1.61)		0 (0-6)		1.5 (1.77)		1 (0-4)		1.04 (1.48)	0 (0-8)	0.07	0.2849	0.427

The correlation coefficient, rho, ranges from -1 to +1. Where 1= perfect positive correlation, 0=no correlation, -1 = perfect negative (inverse) correlation. *Statistical significance at p-value ≤ 0.05.

**Figure 3.**
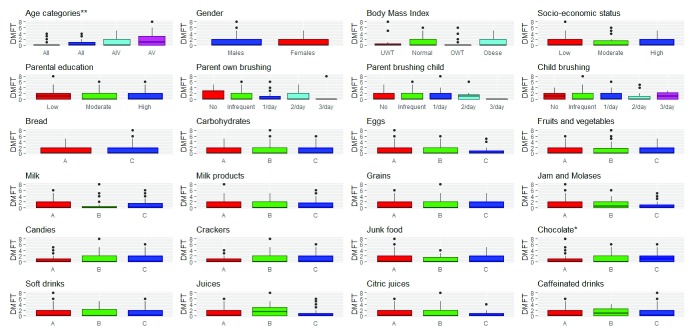
DMFT and different risk factors - N= 369: *+ve correlations; ** inverse correlations; A *: ≤ 2 times/week* B *: 3–6 times/week* C *: 1–6 times/day*.

Males and children within the normal range for BMI had the highest mean DMFT (1.28 ±1.9, 1.15 ±1.52) respectively. The difference in medians was statistically insignificant for gender (p-value>0.05) and statistically significant (p-value=0.0353) for BMI. Correlation coefficient revealed no correlation between gender and BMI with DMFT (Spearman’s rho= -0.05, -0.04, p-value=0.4363, 0.5794).

The highest mean DMFT was for children with high SES and low parental education (1.29 ±1.73, 1.45 ±1.93). The differences in medians were statistically insignificant (p-value>0.05) and correlation coefficients were also insignificant (Spearman’s rho= -0.02, -0.07, p-value=0.7829, 0.2968).

The highest mean DMFT was for children whose parents brush their own teeth three times a day (1.6 ±3.58) and their child’s teeth once a day (1.19 ±1.86) as well as for children who don’t brush their teeth (1.19±1.47). The differences in medians for DMFT were statistically insignificant (p-value>0.05) and there was no correlation between DMFT and any of the biological risk factors.

The highest mean DMFT was for children who consume chocolates 1-6 times per day (1.3±1.56) and children who consume other carbohydrates, eggs, beans, jams, candies, crackers, soda, juices, citric juices and caffeinated drinks 3-6 times per week (1.32±1.95, 1.18±1.78, 1.57±2.47, 1.3±1.72, 1.38±2.16, 1.59±2.43, 1.25±1.75, 1.86±2.23, 1.4±2.23, 1.5±1.77) respectively as well as for children who consume bread, fruits/vegetables, milk, milk products and junk food less than or equal to two times a week (1.25±1.83, 1.23 ±1.68, 1.15±1.55, 1.19 ±1.79). The differences in medians with all the investigated dietary elements were statistically insignificant (p-value>0.05) and there was no correlation between DMFT and any of them except chocolate that had a positive correlation with DMFT (rho=0.16, p-value=0.0178) despite the difference in medians being borderline statistically insignificant (p-value=0.0584).

## Discussion

The high prevalence of dental caries is not only influenced by the biological factors that interact with the causative microorganisms, but it is also associated with socioeconomic, educational conditions and dietary habits ^31^. Dental caries is considered a major public health problem in underdeveloped or developing countries like Egypt. Therefore, investigating the risk factors associated with it has become a major concern to researchers who seek strategies for controlling or preventing the disease. ^31, 32^.

This study investigated the prevalence of dental caries in a wide age range of children because as individuals grow, their dietary needs and habits constantly change. The prevalence of dental caries among Egyptian children was higher in primary dentition (dmft and deft) when compared to permanent dentition (DMFT), this is similar to what has been reported in India ^33, 34^. Deciduous teeth have a higher susceptibility to dental caries due to the lower calcium content and structural differences ^35^. Moreover, caries in the primary dentition could be associated with under nutrition during early childhood. Macro and micro tooth morphology, chemical composition and eruption pattern could be affected by nutrients like vitamin A, vitamin D, calcium and phosphorus ^36^.

In the current study, there was a significant positive correlation between dmft and age, which is in agreement with previous studies conducted in Brazil and Colombia among children aged between 3 to 5 years ^37, 38^. On the other hand, age was inversely correlated with deft in Egyptian children as in mixed dentition period, the maintenance of oral hygiene is difficult due to shedding of primary teeth and pubertal changes.

Investigating the effect of gender on dental caries in the present study revealed that the mean dmft and DMFT of males were higher than those of females while the mean deft of females was slightly higher than that of males. Spearman’s test revealed an inverse correlation between gender and dmft, while no correlation with deft and DMFT. This is similar to a study carried out in Kerala on children aged 12–15 years old, where boys and girls were almost equally affected by caries ^39^, while differs from another cross-sectional study carried on 10-11 years old Italians, where a significant difference was found between DFT of boys (3.20) and DFT of girls (1.96) ^40^. It has been demonstrated that dental caries prevalence switches from male to females with age, where in the 5-year-old age group 47.4% of children with caries were male, while 41.1% were female. On the other hand, in the 12-year-old age group the percentage was inversed (24.1% female versus 20.6% male) ^41^.

Up to date there is a limited evidence clarifying the association between nutritional status and oral health. According to our results, there was no statistically significant correlation between BMI and any of the caries indices. This is inagreement with the findings from previous studies in Taiwan and in Sweden ^42, 43^. Oliveira *et al*. in Brazil ^44^, concluded that underweight children were more likely to have caries which is in accordance with our findings where the highest mean dmft was recorded in underweight children (4.18±4.27). The recorded non significant correlation between DMFT and BMI is consistent with the findings of a previous study among adolescents aged 12 years in public and private schools in São Paulo State ^45^, meanwhile, it is opposite to a study in a German elementary school ^46^ which reported an increase in the DMF with increased BMI. However, in the present investigation a positive association between BMI and deft was recorded, but this was not at a statistically significant level (p=0.9166) which is similarly reported by Elangovan *et al*. ^47^.

Parents play a significant role in the development of their children's oral hygiene habits ^48^. Parental education level, which is directly associated with socioeconomic status ^49^, greatly affects the child's oral health ^50^. In the current study, there was an inverse significant correlation between parental educational level, socioeconomic status and dmft in children which is in accordance with previous studies that reported this correlation in early years ^44, 51, 52^. Low socioeconomic status is usually accompanied by poor dietary habits and unhealthy lifestylesthat contribute to the development of dental caries ^53– 57^. Meanwhile, parents with high socioeconomic and education levels start taking care of their children’s dental health before their second year of life and help them brush their teeth, as reported in a German cross-sectional study ^58^. As the child grows, the parental impact decreases and parents may totally lose their control on the child’s dietary habits and oral hygiene measures. This explains the non-significant correlation between DMFT and parental education, as well as socioeconomic level. Moreover, it has been reported in a study carried out on Indian adolescences that area of residence appears to be a significant determinant for an adolescent to be caries/decay free. Psychosocial and behavioral factors do not mediate the same association ^59^. Since, parents' attitude is the principal social force influencing the child’s development in the early childhood years, therefore parental oral health beliefs and practices may be helpful in the prevention of oral health diseases such as caries ^60^. This is concomitant with our findings where dmft and deft in Egyptian children were correlated to parental brushing to the child and with the child’s own brushing frequency. Children who received oral health education from their parents started to brush their teeth at an earlier age which revealed better dental health ^61^.

Studies that aim to establish the relationship between eating habits and the development of caries preferentially use frequency of food consumption questionnaires such as that employed in our study ^62, 63^. The relationship between the development of carious lesions and dietetic factors has been investigated since the 1940s. It has been suggested that the relationship between sugar intake and the development of carious lesions is currently different from that documented in the past decades, since dental health has improved greatly in the developed countries, with no parallel decrease in the consumption of sugar and cariogenic foods ^64^.

The prevalence of dental caries in view of our results was significantly positively correlated with chocolate, candies and crackers consumption. A direct linkage between sugar intake and caries has been reported previously as cariogenic bacteria grow with the presence of fermentable carbohydrates ^65, 66^. Higher chocolate consumption led to increased caries indices which is consistent with the results from other studies ^67, 68^. Consumption of candies more than once per week, besides insufficient oral hygiene measures have been claimed to be risk factors for caries development in primary and permanent dentition ^69^. Candies remain on the tooth surface for hours and don't have any nutritional value ^70^.

Citric juices were also found to be positively correlated with dental caries in primary teeth in Egyptian children. Hydroxyapatite crystals start to dissolve when the pH reaches 5.5 and enamel begins to be at risk of decalcification. Subsequently, acidic drinks have been reported to play a significant role in the pathogenesis of dental erosion ^71– 75^.

The inverse significant correlation between fruits/vegetables consumption and caries agrees with a study which recorded that dental caries prevalence was higher in non-vegetarians in comparison with vegetarians ^76^. This correlation could be attributed to a lesser tendency for sweets between meals in vegetarians compared with non-vegetarians ^77^. In addition Egyptian children who consumed milk more frequently had lower caries experience. Milk has low cariogenic potential and contains cariostatic factors against dental caries ^78^. Studies showed that milk contains potential caries protective factors as calcium, phosphorus and casein ^79, 80^. The variability of milk consumption manner and other factors may result in a positive association between milk and dental caries occurrence ^80^. This was proven when the frequency of milk consumption did not show a significant association with caries (DMFT).

The insignificant correlations recorded in the current work between all dental caries indices and bread, other carbohydrates, junk food, jam, molasses, honey and juice consumption could be referred to the cross-sectional design of the study. The cross-sectional study may not accurately reflect the true dietary habits of the children before the dental caries occurred since old dietary habits may be responsible for the current development of caries ^81^.

Soda consumption frequency recorded a non-significant correlation with dental caries. This disagrees with past studies which have reported positive correlation between soft drinks and dental caries ^82– 84^. Although sugars in soft drinks lead to drop in the pH of dental plaque and saliva, salivary components can neutralize the acids within 20-30 minutes raising the pH of plaque to its resting level ^85^. Despite the fact that no correlation was found between caffeinated drinks consumption and caries, it was reported that polyphenols in coffee and tea can reduce the cariogenic potential of foods ^86^. Coffee is active against *Streptococcus Mutans*, the organism causing dental caries. Roasted coffee also has anti-adhesive properties. In this manner, it prevents adhesion of *Streptococcus Mutans* to the teeth ^87^.

Finally it could be concluded that in primary dentition, the caries incidence in Egyptian children was positively correlated with candies and crackers while inversely correlated with SES, parental education, brushing frequency of the parent to the child, brushing frequency of the child to him/herself and fruits/vegetables consumption. In permanent dentition DMFT was only significantly positively correlated with age and chocolates.

The World Health Organization emphasizes the need public health solutions for prevention of dental caries. Therefore, the following recommendations based on the results of the current study should be added to the WHO policy measures ^88^ to promote the reduction of dental caries prevalence in Egypt: 1- Candies and crackers should be prohibited for children before 12 years old; 2- Children should be encouraged to eat fruits and vegetables; 3- Awareness campaigns should be carried out to encourage the parents to brush their children’s teeth and to encourage the children to brush their own teeth.

The non-significant differences and lack of correlations between some caries indices and risk factors could be attributed to the small sample size, with a larger set of samples they may have reached statistical significance. In addition, a larger population from different governorates may have allowed broader diversity for better representation for Egyptian population.

## Data availability

Underlying data is available from Figshare.

Figshare: Dataset 1. Raw data for caries incidence in correlation to risk factors in Egyptian children. https://doi.org/10.6084/m9.figshare.7445843.v1 ^89^

License: CC0 1.0 Universal (CC0 1.0) Public Domain Dedication

### Extended data

The study questionnaire is available from Figshare.

Figshare: Extended data. Questionnaire for children. https://doi.org/10.6084/m9.figshare.7392170.v3 ^90^

License: CC0 1.0 Universal (CC0 1.0) Public Domain Dedication

## References

[1] Selwitz RH Ismail AI Pitts NB: Dental caries. *Lancet.* 2007;369(9555):51–59. 10.1016/S0140-6736(07)60031-2 17208642

[2] GBD 2015 Disease and Injury Incidence and Prevalence Collaborators: Global, regional, and national incidence, prevalence, and years lived with disability for 310 diseases and injuries, 1990-2015: a systematic analysis for the Global Burden of Disease Study 2015. *Lancet.* 2016;388(10053):1545–1602. 10.1016/S0140-6736(16)31678-6 27733282 PMC5055577

[3] Que G Hou W: [Epidemiological investigation on deciduous dental caries among children aged 2 approximately 4 years of Kaifu district in Changsha city]. *Zhong Nan Da Xue Xue Bao Yi Xue Ban.* 2009;34(1):76–80. 19197133

[4] Armfield JM Slade GD Spencer AJ: Socioeconomic differences in children's dental health: the Child Dental Health Survey, Australia 2001. AIHW Dental Statistics and Research Unit.2006 Reference Source

[5] Poureslami HR Van Amerongen WE: Early Childhood Caries (ECC): an infectious transmissible oral disease. *Indian J Pediatr.* 2009;76(2):191–194. 10.1007/s12098-008-0216-1 19082537

[6] Albino J Tiwari T: Preventing Childhood Caries: A Review of Recent Behavioral Research. *J Dent Res.* 2016;95(1):35–42. 10.1177/0022034515609034 26438210 PMC4700662

[7] Petersen PE Kjoller M Christensen LB: Changing dentate status of adults, use of dental health services, and achievement of national dental health goals in Denmark by the year 2000. *J Public Health Dent.* 2004;64(3):127–135. 10.1111/j.1752-7325.2004.tb02742.x 15341135

[8] Widström E Eaton KA: Oral healthcare systems in the extended European union. *Oral Health Prev Dent.* 2004;2(3):155–194. 15641621

[9] Petersen PE: Challenges to improvement of oral health in the 21st century--the approach of the WHO Global Oral Health Programme. *Int Dent J.* 2004;54(6 Suppl 1):329–343. 10.1111/j.1875-595X.2004.tb00009.x 15631094

[10] Leake J Jozzy S Uswak G: Severe dental caries, impacts and determinants among children 2-6 years of age in Inuvik Region, Northwest Territories, Canada. *J Can Dent Assoc.* 2008;74(6):519. 18644236

[11] Zahran M: Dental health of primary school children in Egypt: an incremental care program. *Egypt Dent J.* 1973;19(2):265–276. 4524097

[12] Zahran M: The pattern of dental caries in Egyptian school children. *Egypt Dent J.* 1974;20(4):13–26. 4157209

[13] Abdel-Azim MM: Restorative dental status and restorative dental needs among a sample of Egyptian dental students. *Egypt Dent J.* 1979;25(3):269–278. 299157

[14] el-Beheri S: Dental caries among Egyptian school children of high and low socio-economic level. *Egypt Dent J.* 1986;32(3):255–263. 3464397

[15] Wissa AA Zahran MA: Evaluation of governmental dental health services in rural health centers in Egypt. *Community Dent Oral Epidemiol.* 1988;16(1):16–18. 10.1111/j.1600-0528.1988.tb00546.x 3422611

[16] http://www.emro.who.int/egy/egypt-events/results-of-epidemiological-study-on-oral-health-status-released.html.2014.

[17] Abou El-Yazeed M Rashed M El sayed M: Dental Caries Prevalence among a group of Egyptian Nurseries Children. *Life Sci J.* 2011;8(1):412–419. Reference Source

[18] Hamila NAA: Early childhood caries and certain risk factors in a sample of children 1-3.5 years in Tanta. *Dentistry.* 2013;4:180 10.4172/2161-1122.1000180

[19] Mobarak EH Shabayek MM Mulder J: Caries experience of Egyptian adolescents: does the atraumatic restorative treatment approach offer a solution? *Med Princ Pract.* 2011;20(6):545–9. 10.1159/000329790 21986013

[20] Daniel WW: Biostatistics: A Foundation for Analysis in the Health Sciences.7th edition. New York: John Wiley & Sons.1999 Reference Source

[21] Veerasamy A Kirk R Gage J: Epidemiology of dental caries among adolescents in Tamil Nadu, India. *Int Dent J.* 2016;66(3):169–77. 10.1111/idj.12216 26825051 PMC9376636

[22] Quach A Laemmle-Ruff IL Polizzi T: Gaps in smiles and services: a cross-sectional study of dental caries in refugee-background children. *BMC Oral Health.* 2015;15(1):10. 10.1186/1472-6831-15-10 25608733 PMC4324800

[23] World Health Organization: Expert Committee on Physical Status: the use and interpretation of anthropometry physical status. WHO Techniques Report Series, v 854. Geneva: World Health Organization.1995 Reference Source 8594834

[24] Martins RJ Moimaz SA Silva MR: Body mass index, dental caries and sugar intake in 2-5 year-old preschoolers. *Braz J Oral Sci.* 2014;13(3):209–212. 10.1590/1677-3225v13n3a09

[25] Edalat A Abbaszadeh M Eesvandi M: The Relationship of Severe Early Childhood Caries and Body Mass Index in a Group of 3- to 6-year-old Children in Shiraz. *J Dent (Shiraz).* 2014;15(2):68–73. 24883343 PMC4033086

[26] Kuczmarski RJ Ogden CL Grummer-Strawn LM: CDC growth charts: United States. *Adv Data.* 2000;8(314):1–27. 11183293

[27] Jokić NI Bakarčić D Janković S: Dental caries experience in croatian school children in Primorsko-Goranska county. *Cent Eur J Public Health.* 2013;21(1):39–42. 10.21101/cejph.a3752 23741899

[28] World Health Organization: Oral Health Surveys: Basic Methods.World Health Organization, Geneva, Switzerland.1997 Reference Source

[29] World Health Organization: Oral health surveys: Basic methods. 5th ed., Geneva, Switzerland, World Health Organization.2013 Reference Source

[30] El-Gilany A El-Wehady A El-Wasify M: Updating and validation of the socioeconomic status scale for health research in Egypt. *East Mediterr Health J.* 2012;18(9):962–968. 10.26719/2012.18.9.962 23057390

[31] McGrady MG Ellwood RP Goodwin M: Adolescents' perceptions of the aesthetic impact of dental fluorosis vs. other dental conditions in areas with and without water fluoridation. *BMC Oral Health.* 2012;12(1):4. 10.1186/1472-6831-12-4 22325055 PMC3306760

[32] Cypriano S Hoffmann RH de Sousa Mda L: Dental caries experience in 12-year-old schoolchildren in southeastern Brazil. *J Appl Oral Sci.* 2008;16(4):286–292. 10.1590/S1678-77572008000400011 19089262 PMC4327539

[33] Goyal A Gauba K Chawla HS: Epidemiology of dental caries in Chandigarh school children and trends over the last 25 years. *J Indian Soc Pedod Prev Dent.* 2007;25(3):115–118. 10.4103/0970-4388.36559 17951925

[34] Sudha P Bhasin S Anegundi RT: Prevalence of dental caries among 5-13-year-old children of Mangalore city. *J Indian Soc Pedod Prev Dent.* 2005;23(2):74–79. 10.4103/0970-4388.16446 16012209

[35] Jain A Jain V Suri SM: Prevalence of dental caries in male children from 3 to 14 years of age of Bundelkhand region, India. *Int J Community Med Public Health.* 2016;3(4):787–790. 10.18203/2394-6040.ijcmph20160730

[36] Psoter WJ Reid BC Katz RV: Malnutrition and dental caries: a review of the literature. *Caries Res.* 2005;39(6):441–447. 10.1159/000088178 16251787 PMC1362953

[37] Xavier A Bastos RDS Arakawa AM: Correlation between dental caries and nutritional status: preschool children in a Brazilian municipality. *Revista de Odontologia da UNESP.* 2013;42(5):378–383. 10.1590/S1807-25772013000500010

[38] Ojeda JC Llanos LS: Caries prevalence of preschool age children in community homes of the Cauca Valle and related social factors. *Revista Odontológica Mexicana.* 2017;21(4):e221–e226. 10.1016/j.rodmex.2018.01.011

[39] Jose A Joseph MR: Prevalence of dental health problems among school going children in rural Kerala. *J Indian Soc Pedod Prev Dent.* 2003;21(4):147–51. 14765615

[40] Migale D Barbato E Bossù M: Oral health and malocclusion in 10-to-11 years-old children in southern Italy. *Eur J Paediatr Dent.* 2009;10(1):13–18. 19364240

[41] Saravanan S Anuradha KP Bhaskar DJ: Prevalence of dental caries and treatment needs among school going children of Pondicherry, India. *J Indian Soc Pedod Prev Dent.* 2003;21(1):1–12. 12885002

[42] Alm A Isaksson H Fahraeus C: BMI status in Swedish children and young adults in relation to caries prevalence. *Swed Dent J.* 2011;35(1):1–8. 21591594

[43] Chen W Chen P Chen SC: Lack of association between obesity and dental caries in three-year-old children. *Zhonghua Min Guo Xiao Er Ke Yi Xue Hui Za Zhi.* 1998;39(2):109–111. 9599900

[44] Oliveira LB Sheiham A Bönecker M: Exploring the association of dental caries with social factors and nutritional status in Brazilian preschool children. *Eur J Oral Sci.* 2008;116(1):37–43. 10.1111/j.1600-0722.2007.00507.x 18186730

[45] Sales-Peres SH Goya S Sant’Anna RM: [Prevalence of overweight and obesity, and associated factors in adolescents, at the central west area of the state São Paulo (SP, Brazil)]. *Cien Saude Colet.* 2010;15 Suppl 2:3175–3184. 10.1590/S1413-81232010000800022 21049158

[46] Willerhausen B Blettner M Kasaj A: Association between body mass index and dental health in 1,290 children of elementary schools in a German city. *Clin Oral Investig.* 2007;11(3):195–200. 10.1007/s00784-007-0103-6 17294228

[47] Elangovan A Mungara J Joseph E: Exploring the relation between body mass index, diet, and dental caries among 6-12-year-old children. *J Indian Soc Pedod Prev Dent.* 2012;30(4):293–300. 10.4103/0970-4388.108924 23514680

[48] Saldūnaitė K Bendoraitienė EA Slabšinskienė E: The role of parental education and socioeconomic status in dental caries prevention among Lithuanian children. *Medicina (Kaunas).* 2014;50(3):156–161. 10.1016/j.medici.2014.07.003 25323543

[49] Pizzo G Piscopo MR Matranga D: Prevalence and socio-behavioral determinants of dental caries in Sicilian schoolchildren. *Med Sci Monit.* 2010;16(10):PH83–9. 20885361

[50] Koposova N Widström E Eisemann M: Oral health and quality of life in Norwegian and Russian school children: A pilot study. *Stomatologia.* 2010;12(1):10–16. 20440091

[51] Kato H Tanaka K Shimizu K: Parental occupations, educational levels, and income and prevalence of dental caries in 3-year-old Japanese children. *Environ Health Prev Med.* 2017;22(1):80. 10.1186/s12199-017-0688-6 29237397 PMC5729505

[52] Hooley M Skouteris H Boganin C: Parental influence and the development of dental caries in children aged 0-6 years: a systematic review of the literature. *J Dent.* 2012;40(11):873–85. 10.1016/j.jdent.2012.07.013 22842202

[53] Marshall TA Eichenberger-Glilmore JM Broffitt BA: Dental caries and childhood obesity: roles of diet and socioeconomic status. *Community Dent Oral Epidemiol.* 2007;35(6):449–458. 10.1111/j.1600-0528.2006.00353.x 18039286

[54] Jürgensen N Petersen PE: Oral health and the impact of socio-behavioural factors in a cross sectional survey of 12-year old school children in Laos. *BMC Oral Health.* 2009;9(1):29. 10.1186/1472-6831-9-29 19917089 PMC2781791

[55] Bruno-Ambrosius K Swanholm G Twetman S: Eating habits, smoking and toothbrushing in relation to dental caries: a 3-year study in Swedish female teenagers. *Int J Paediatr Dent.* 2005;15(3):190–6. 10.1111/j.1365-263X.2005.00621.x 15854115

[56] Ayele FA Taye BW Ayele TA: Predictors of dental caries among children 7-14 years old in Northwest Ethiopia: a community based cross-sectional study. *BMC Oral Health.* 2013;13:7. 10.1186/1472-6831-13-7 23331467 PMC3554509

[57] Mafuvadze BT Mahachi L Mafuvadze B: Dental caries and oral health practice among 12 year old school children from low socio-economic status background in Zimbabwe. *Pan Afr Med J.* 2013;14:164. 10.11604/pamj.2013.14.164.2399 23819006 PMC3696470

[58] Pieper K Dressler S Heinzel-Gutenbrunner M: The influence of social status on pre-school children’s eating habits, caries experience and caries prevention behavior. *Int J Public Health.* 2012;57(1):207–15. 10.1007/s00038-011-0291-3 21912945

[59] Mathur MR Tsakos G Millett C: Socioeconomic inequalities in dental caries and their determinants in adolescents in New Delhi, India. *BMJ Open.* 2014;4(12):e006391. 10.1136/bmjopen-2014-006391 25500618 PMC4267077

[60] Hooley M Skouteris H Boganin C: Parental influence and the development of dental caries in children aged 0-6 years: a systematic review of the literature. *J Dent.* 2012;40(11):873–885. 10.1016/j.jdent.2012.07.013 22842202

[61] Boka V Trikaliotis A Kotsanos N: Dental caries and oral health-related factors in a sample of Greek preschool children. *Eur Arch Paediatric Dent.* 2013;14(6):363–368. 10.1007/s40368-013-0097-5 24277257

[62] Llena C Forner L: Dietary habits in a child population in relation to caries experience. *Caries Res.* 2008;42(5):387–393. 10.1159/000154784 18781067

[63] Llena Puy C Forner L: Dietary habits of a school population and implications for oral health. *Minerva Stomatol.* 2010;59(4):173–180. 20360665

[64] Serra-Majem L García Closas R Ramón JM: Dietary habits and dental caries in a population of Spanish schoolchildren with low levels of caries experience. *Caries Res.* 1993;27(6):488–494. 10.1159/000261586 8281564

[65] Çolak H Dülgergil ÇT Dalli M: Early childhood caries update: A review of causes, diagnoses, and treatments. *J Nat Sci Biol Med.* 2013;4(1):29–38. 10.4103/0976-9668.107257 23633832 PMC3633299

[66] Sheiham AJ James WP: A reappraisal of the quantitative relationship between sugar intake and dental caries: the need for new criteria for developing goals for sugar intake. *BMC Public Health.* 2014;14:863. 10.1186/1471-2458-14-863 25228012 PMC4168053

[67] Doichinova L Bakardjiev P Peneva M: Assessment of food habits in children aged 6-12 years and the risk of caries. *Biotechnol Biotechnol Equip.* 2015;29(1):200–204. 10.1080/13102818.2014.989180 26019634 PMC4433830

[68] Mafuvadze BT Mahachi L Mafuvadze B: Dental caries and oral health practice among 12 year old school children from low socio-economic status background in Zimbabwe. *Pan Afr Med J.* 2013;14:164. 10.11604/pamj.2013.14.164.2399 23819006 PMC3696470

[69] Mobley C Marshall TA Milgrom P: The contribution of dietary factors to dental caries and disparities in caries. *Acad Pediatr.* 2009;9(6):410–414. 10.1016/j.acap.2009.09.008 19945075 PMC2862385

[70] Almasi A Rahimiforoushani A Eshraghian MR: Effect of Nutritional Habits on Dental Caries in Permanent Dentition among Schoolchildren Aged 10-12 Years: A Zero-Inflated Generalized Poisson Regression Model Approach. *Iran J Public Health.* 2016;45(3):353–61. 27141498 PMC4851750

[71] Mathew T Casamassimo PS Hayes JR: Relationship between sports drinks and dental erosion in 304 university athletes in Columbus, Ohio, USA. *Caries Res.* 2002;36(4):281–287. 10.1159/000063927 12218278

[72] Al-Majed I Maguire A Murray JJ: Risk factors for dental erosion in 5-6 year old and 12-14 year old boys in Saudi Arabia. *Community Dent Oral Epidemiol.* 2002;30(1):38–46. 10.1034/j.1600-0528.2002.300106.x 11918574

[73] Rees J Loyn T McAndrew R: The acidic and erosive potential of five sports drinks. *Eur J Prosthodont Restor Dent.* 2005;13(4):186–190. 16411577

[74] Tahmassebi JF Duggal MS Malik-Kotru G: Soft drinks and dental health: a review of the current literature. *J Dent.* 2006;34(1):2–11. 10.1016/j.jdent.2004.11.006 16157439

[75] Soares PV Tolentino AB Machado AC: Sports dentistry: a perspective for the future. *Rev Bras Educ Fís Esporte.* 2014;28(2):351–358. 10.1590/1807-55092014000200351

[76] Shah N Sundaram KR: Impact of socio-demographic variables, oral hygiene practices, oral habits and diet on dental caries experience of Indian elderly: a community‐based study. *Gerodontology.* 2004;21(1):43–50. 10.1111/j.1741-2358.2004.00010.x 15074539

[77] Sherfudhin H Abdullah A Shaik H: Some aspects of dental health in young adult Indian vegetarians. A pilot study. *Acta Odontol Scand.* 1996;54(1):44–48. 10.3109/00016359609003508 8669240

[78] Johansson I: Milk and dairy products: Possible effects on dental health. *Food Nutr Res.* 2002;46(3):119–122. 10.1080/11026480260363242

[79] Moynihan P Petersen PE: Diet, nutrition and the prevention of dental diseases. *Public Health Nutr.* 2004;7(1A):201–226. 10.1079/PHN2003589 14972061

[80] Lim S Sohn W Burt BA: Cariogenicity of soft drinks, milk and fruit juice in low-income african-american children: a longitudinal study. *J Am Dent Assoc.* 2008;139(7):959–967, quiz 995. 10.14219/jada.archive.2008.0283 18594082

[81] Zahara AM Ili MN Yahya NA: Dietary habits and dental caries occurrence among young children: Does the relationship still exist? *Malaysian J Med Health Sci.* 2013;9(1):9–20. Reference Source

[82] Levy SM Warren JJ Broffitt B: Fluoride, beverages and dental caries in the primary dentition. *Caries Res.* 2003;37(3):157–165. 10.1159/000070438 12740537

[83] Sohn W Burt BA Sowers MR: Carbonated soft drinks and dental caries in the primary dentition. *J Dent Res.* 2006;85(3):262–266. 10.1177/154405910608500311 16498075

[84] Stevens A Hamel C Singh K: Do sugar-sweetened beverages cause adverse health outcomes in children? A systematic review protocol. *Syst Rev.* 2014;3(1):96. 10.1186/2046-4053-3-96 25192945 PMC4160918

[85] Ismail AI Burt BA Eklund SA: The cariogenicity of soft drinks in the United States. *J Am Dent Assoc.* 1984;109(2):241–245. 10.14219/jada.archive.1984.0346 6590604

[86] Ooshima T Minami T Aono W: Oolong tea polyphenols inhibit experimental dental caries in SPF rats infected with mutans streptococci. *Caries Res.* 1993;27(2):124–129. 10.1159/000261529 8319255

[87] Daglia M Racchi M Papetti A: *In vitro* and *ex vivo* antihydroxyl radical activity of green and roasted coffee. *J Agric Food Chem.* 2004;52(6):1700–4. 10.1021/jf030298n 15030233

[88] World Health Organization (WHO): Sugars intake for adults and children.Geneva: WHO,2015; accessed 17 September 2017. Reference Source

[89] Abbass M: Raw data for caries incidence in correlation to risk factors in Egyptian children.2018 10.6084/m9.figshare.7445843.v1

[90] Abbass M: questionnaire for children.pdf.2018 10.6084/m9.figshare.7392170.v3

