# Supplementary material for: The prevalence of dental caries among Egyptian children and adolescences and its association with age, socioeconomic status, dietary habits and other risk factors. A cross-sectional study
Source: F1000Res. 2019 Jan 3;8:8. [Version 1] doi: 10.12688/f1000research.17047.1 (PMC6396843; doi:10.12688/f1000research.17047.1)
Supplement: Peer review history

# Peer review: Reviewer response for version 1

**DOI:** 10.5256/f1000research.18637.r44367

**Published:** 2019-02-27

**Authors:** Ali Arabi

**Affiliations:** 1 Faculty of Medicine, University of Khartoum, Khartoum, Sudan

- This article strongly supports the research dental health and in relation to oral health and dietary habits. But the tittle is too long 
- Using different risk factors with no good outcomes 
- Oral health is a key factors not well addressed in introduction especially guidelines for small children and adolescents 
- Very detailed about dental caries but few about oral health and dietary habits 
- Research questions not all will addressed in methodology 
- The methodology is adequate but Lacks the methodology of dietary habits is it.
- Diet is a major lifestyle-related risk factor of various chronic diseases. Dietary intake can be assessed by subjective report and objective observation. Subjective assessment is possible using open-ended surveys such as dietary recalls or records, or using closed-ended surveys including food frequency questionnaires. Each method has inherent strengths and limitation  not clear which method used need clarifications 

However, there are a few issues that need to be addressed in the results 

1. Table 3, 4 and 5 are confusing…. So data is reported in mean (SD) and median…with range or IQ range it’s not clear in the table caption…. Please clarify that.. A typo error in the dietary habits. please correct.  
2. Also reporting the Mean (SD) is not suitable for all the data reported except for age.. so reporting that value add more to the confusion and increases the table sizes with no value……reporting the frequency is a must.
3. K-W test when significant please report the pairwise comparison

- References are up-to-date 

Question to author: 

- Could the title of paper changed to:

The prevalence of dental caries among Egyptian young children and adolescences and its association with oral health and dietary habits because age and socioeconomic factors and other factors has no outcomes in the study paper 

 In the recommendations, should be age related young children should different from from adolescence because of different dietary habits and oral health and age group

I have read this submission. I believe that I have an appropriate level of expertise to confirm that it is of an acceptable scientific standard, however I have significant reservations, as outlined above.

# Peer review: Reviewer response for version 1

**DOI:** 10.5256/f1000research.18637.r44369

**Published:** 2019-02-20

**Authors:** Rania Mossad Hassan

**Affiliations:** 1 Faculty of Dentistry, Ain Shams University, Cairo, Egypt; 2 Badr University in Cairo, Badr City, Egypt

This study investigated the dental caries prevalence among Egyptian children and adolescents in association with different risk factors.

 At the beginning, I would like to congratulate the authors for attempting to undertake this work, due to data shortage or even lack of data regarding this subject. I found it very interesting and of valuable additional knowledge.

 The manuscript is well-written and well-structured. The relationships between different caries indices, SES and parental education haven't been investigated before among Egyptian children. Moreover, this is the first work that deals with the correlation between cariogenic and non cariogenic dietary elements with the caries indices among Egyptian children.

 The discussion is well written covering all the study results. The data from this work could be used to make regression analysis to identify the causative risk factors.

 However, the paper is clear substantially easy to read but still there are suggested minor comments the author could deal with, or discuss for additional impact.

1. The results are given in full details, the great amount of data make it difficult for the reader to understand some relations.
2. The age ranges differed, as they were  3- 12 and  13-18 years  when mentioned  in subjects and methods, meanwhile in data collection, the age groups were classified into 3-5, 5-7, 7-9, 9-12 and 12-18 years. On the other hand, in the tables, the age groups were 3-4, 5-6, 7-8, 9-11,12-17 years. So, they are to be corrected.

I have read this submission. I believe that I have an appropriate level of expertise to confirm that it is of an acceptable scientific standard.

# Peer review: Reviewer response for version 1

**DOI:** 10.5256/f1000research.18637.r44056

**Published:** 2019-02-08

**Authors:** Mauro Henrique Nogueira Guimarães Abreu

**Affiliations:** 1 Department of Community and Preventive Dentistry, Federal University of Minas Gerais (UFMG), Belo Horizonte, Brazil

This study focused on dental caries prevalence and some associated factors among Egyptian children and adolescents. The study has a good rationale and reasonable theoretical framework. There are some issues and problems that should be corrected by authors:

1. The term “correlation” should be modified to “association” in all sections of the manuscript. The term “correlation” should be maintained only where Spearman’s Correlation Coefficient was correctly calculated.
2. The authors should present a good justification for not using d mft index.
3. “Subjects who, in the opinion of the Investigator, may be non-compliant with study procedures.” – This criterion is quite subjective.
4. In the sample size calculation, the authors should clearly point out that they use the prevalence of “60%”. The most serious issue is how the individuals were selected (simple random sample? Cluster random sample? Another sampling strategy?)
5. There are some English language errors that must be corrected. I strongly recommend that a professional native English be contacted.
6. Statistical analysis is worrisome. Why authors use the Shapiro-Wilk test? Why did they not use the Kolmogorov-Smirnov test? The indication of correlation analysis for comparing nominal and quantitative (Eg. Gender and dmft) variables is also incorrect.
7. The influence of age in dental caries is not a new finding in the literature. Therefore, authors should stratify the age groups with similar prevalence and severity of dental caries in order to perform a better and rigorous analysis.
8. The authors should perform multiple regression models for all dependent variables.
9. Without these previously corrections, it is not possible to evaluate the results, discussion, and conclusions.

I have read this submission. I believe that I have an appropriate level of expertise to confirm that it is of an acceptable scientific standard, however I have significant reservations, as outlined above.
